# The Rabs: A family at the root of metazoan evolution

**DOI:** 10.1186/1741-7007-10-68

**Published:** 2012-08-08

**Authors:** Harald Stenmark

**Affiliations:** 1 Centre for Cancer Biomedicine, Faculty of Medicine, Oslo University Hospital, Montebello, N-0310 Oslo, Norway; 2 Department of Biochemistry, Institute for Cancer Research, Oslo University Hospital, Montebello, N-0310 Oslo, Norway

## Abstract

Eukaryotic cells are distinguished by their compartmentalization into membrane-enclosed organelles that exchange membranes and content in a highly ordered manner. Central in defining membrane identity are the Rabs, a large family of small GTPases that localize to distinct membranes and recruit specific regulators of membrane traffic. Two recent papers, including one by Klöpper *et al*. in *BMC Biology*, present phylogenomic evidence that the Rab repertoire was established very early in eukaryotic evolution, and correlates with interspecies variations in organelles.

See research article http://www.biomedcentral.com/1741-7007/10/71

## Rabs as regulators of intracellular membrane traffic

**Figure 1:**
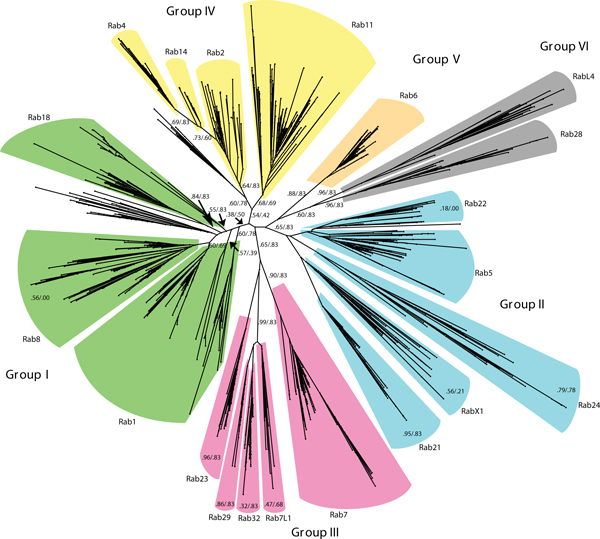
**Evolutionary tree depicting the relationships of the different Rab families proposed to have been present in the last eukaryotic common ancestor (LECA)**. (Figure reproduced from Figure 1 of Klöpper *et al*. [6].).

The Rab family is by far the largest among the small GTPases, with more than 60 members in humans [1]. Originally identified as regulators of membrane traffic in yeast, the Rabs have been found to regulate membrane traffic in a large number of species ranging from amoeba and yeast to plants, nematodes, insects, and humans [2]. Different family members are known to localize to distinct organelle membranes in a reversible manner, and several regulators of Rab membrane association are known, including proteins that mediate the prenylation of one or two carboxy-terminal cysteine residues of the Rabs [1,3]. There is no single mechanism by which Rab GTPases direct membrane traffic, although the switching between GTP- and GDP-bound forms (promoted by specific guanine nucleotide exchange factors and GTPase-activating proteins, respectively) is central to their function [1]. In their GTP-bound form, Rab GTPases recruit effector proteins of various types, including membrane tethering factors, phosphatases, kinases, and cytoskeletal motors. The recruitment of these effectors to specific membranes at specific time points is the principal way by which Rabs control membrane dynamics and identity [1,3]. Because this specificity requires the existence of many different Rabs that recruit distinct effectors, the issue of identifying and classifying different Rab family members in various organisms has excited considerable interest, and several excellent papers on this issue have already been published [2,4,5]. Klöpper *et al*. now present in *BMC Biology *the most comprehensive phylogenetic study of Rabs so far [6]. This study shows that, at the earliest identifiable point in eukaryotic evolution, the so-called last eukaryotic common ancestor (LECA) [7], there was already a remarkably large number of different Rabs. Interestingly, Klöpper *et al*. were able to classify the LECA Rabs into six supergroups (Figure 1) that can be traced through evolution. This analysis may prove very valuable, not only for those studying Rabs, but also for all those interested in eukaryotic evolution.

## Tracing Rab evolution - a complex task

The era of whole-genome sequencing has offered new avenues for studying evolution at the molecular level, and Klöpper *et al*. took advantage of publicly available information on genome projects for as many as 384 different species, plus more than 200 species for which sufficient sequence information is known in other ways. Identifying Rabs by bioinformatics is no trivial task, however, since different Rabs show only regional sequence similarities and there are several hypervariable sequences [4]. Adding to this complexity, other small GTPases show partial sequence similarity to Rabs, and it is difficult to sort out whether borderline cases represent genuine Rabs or not. The authors therefore started with a limited set of sequences (about 500 from 21 species) and used a hidden Markov model (a mathematical model favored by many bioinformaticists for comparing sequences in a relatively unbiased manner) in order to extract a core Rab motif that was used to screen the larger sample set, which contained more than 7,600 sequences from Rabs representing all major eukaryotic phyla. Out of this extensive analysis the authors identified a set of 20 basic Rab types, and by testing several hypotheses for the placement of the LECA in the root of the eukaryotic tree of life, the authors concluded that the LECA is most likely to have contained all these 20 Rabs. This is a surprisingly high number, but it correlates well with a recent study that used a smaller query set and a somewhat different methodology, and which estimated the number of different Rabs in the LECA to be up to 23 [5]. The two studies thus agree that the Rab repertoire developed at a very early point in eukaryotic history.

## Functional diversification of Rabs

**Figure 2:**
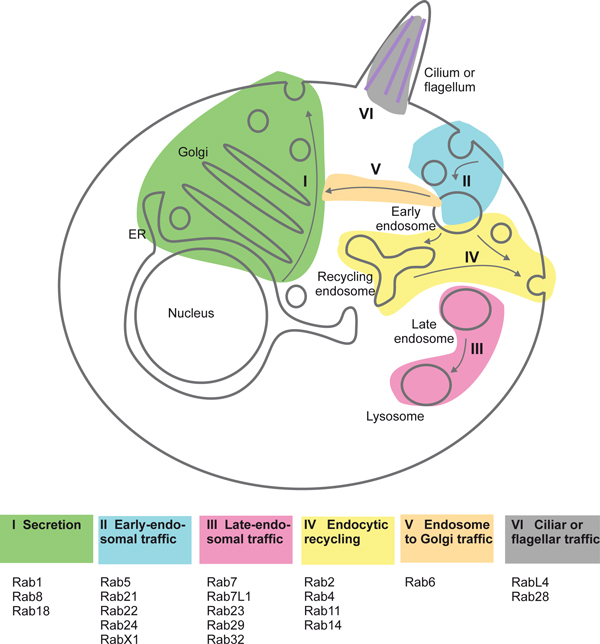
**The LECA Rabs and their proposed functions**. A schematic LECA cell is shown with the routes controlled by the six Rab supergroups defined by Klöpper *et al*. [6] color-coded. The Rabs belonging to the respective groups are indicated below. Even though most of the Rabs have been retained in human cells, two have been lost (RabX1 and Rab29). Note that even if there is a good match between the phylogenetic grouping and the cellular pathways controlled by the respective Rabs, there are some possible misfits: Rab24 (group II) has been found to regulate autophagy; Rab23 (group III) has been described to regulate ciliary functions; Rab2 (group IV) was identified as a regulator of traffic in the early biosynthetic pathway; Rab6 (group V) has, in addition to regulating endosome-to-Golgi transport, also been implicated in intra-Golgi trafficking (see references in [1]). ER, endoplasmic reticulum.

Early studies in yeast and mammalian cell culture established that distinct Rabs localize to distinct membranes and control distinct membrane transport pathways [8-10], and from numerous studies in various organisms we now have a reasonably clear picture (at least for most of the Rabs) of which pathways are controlled by which Rabs [1]. Importantly, Rab orthologs in such different species as yeast, plants, insects and humans have been found to perform functions that are highly (albeit not completely) related. In this context it is interesting to note that the six Rab supergroups identified in the LECA by Klöpper *et al*. largely correspond to six different routes of membrane traffic (Figure 2): secretion (group I), early-endosomal traffic (group II), late-endosomal traffic (group III), recycling from endosomes to the cell surface (group IV), 'retrograde' transport from endosomes to Golgi apparatus (group V); and traffic associated with cilia or flagella (group VI). This is consistent with the view that the LECA was equipped with all these major trafficking routes.

## Rab evolution as indicator of organelle plasticity

When comparing the Rab repertoires of different species, it is conspicuous that some Rabs have been selectively lost during evolution of various species whereas others have expanded and diversified [5,6]. For instance, a large number of new Rabs in group I have appeared in metazoans, which may reflect the fact that metazoans are multicellular and contain polarized cells, which requires a diversified set of exocytic routes [6]. In fact, the gains and losses of Rabs can to a large extent be correlated with the numbers and types of organelles in the various species, a correlation that has not been found for other regulators of membrane traffic such as coat proteins, vesicle tethers and proteins directly involved in membrane fusion [6]. This opens the possibility that gains, diversifications and losses of Rabs may have been the driving forces for organelle plasticity during evolution. Given the importance of organelle plasticity for species diversification and evolution, the recent findings should place Rabs among the favorite proteins for evolutionary biologists.
